# Baicalin Alleviates Short-Term Lincomycin-Induced Intestinal and Liver Injury and Inflammation in Infant Mice

**DOI:** 10.3390/ijms23116072

**Published:** 2022-05-28

**Authors:** Shunfen Zhang, Ruqing Zhong, Shanlong Tang, Hui Han, Liang Chen, Hongfu Zhang

**Affiliations:** State Key Laboratory of Animal Nutrition, Institute of Animal Science, Chinese Academy of Agricultural Sciences, Beijing 100193, China; zhangshunfen614@163.com (S.Z.); zhongruqing@caas.cn (R.Z.); long18763897938@163.com (S.T.); hanhui16@mails.ucas.ac.cn (H.H.)

**Keywords:** baicalin, lincomycin, gut-liver axis, RNA-seq, injury repair, microbiota, T-helper cell differentiation

## Abstract

The adverse effects of short-term megadose of antibiotics exposure on the gastrointestinal and liver tissue reactions in young children have been reported. Antibiotic-induced intestinal and liver reactions are usually unpredictable and present a poorly understood pathogenesis. It is, therefore, necessary to develop strategies for reducing the adverse effects of antibiotics. Studies on the harm and rescue measures of antibiotics from the perspective of the gut–liver system are lacking. Here, we demonstrate that lincomycin exposure reduced body weight, disrupted the composition of gut microbiota and intestinal morphology, triggered immune-mediated injury and inflammation, caused liver dysfunction, and affected lipid metabolism. However, baicalin administration attenuated the lincomycin-induced changes. Transcriptome analysis showed that baicalin improved immunity in mice, as evidenced by the decreased levels of intestinal inflammatory cytokines and expression of genes that regulate Th1, Th2, and Th17 cell differentiation, and inhibited mucin type O-glycan biosynthesis pathways. In addition, baicalin improved liver function by upregulating the expression of genes involved in bile acid secretion and lipid degradation, and downregulating genes involved in lipid synthesis in lincomycin-treated mice. Bile acids can regulate intestinal immunity and strengthen hepatoenteric circulation. In addition, baicalin also improved anti-inflammatory bacteria abundance (*Blautia* and *Coprobacillus*) and reduced pathogenic bacteria abundance (*Proteobacteria*, *Klebsiella*, and *Citrobacter*) in lincomycin-treated mice. Thus, baicalin can ameliorate antibiotic-induced injury and its associated complications such as liver disease.

## 1. Introduction

Developed countries have reduced the use of antibiotics in pediatric clinical medicine [1]. However, to achieve ideal treatment effects, megadose antibiotics use during infections remains important. Therefore, there is a need to elucidate on the effects of short-term megadose antibiotic exposure on children. Lincomycin and related antibiotics, such as clindamycin, are widely used as prophylactic measure in gastrointestinal operations, including in colorectal cancer resection [2]. Long-term lincomycin applications are associated with various side effects, including diarrhea, inflammatory bowel diseases, pseudomembranous colitis, and liver dysfunctions [3]. Often, these effects are difficult to be detected and controlled, and persist as “secondary damage” to the patient, sometimes leading to severe sepsis [4]. Short-term (one week) lincomycin exposure leads to intestinal microbial disturbance and inflammation [5]. Antibiotics are also the most common drug class causing liver injury [6]. Antibiotic-induced hepatotoxic reactions are usually idiosyncratic, unpredictable, and present a poorly understood pathogenesis [7]. Antibiotic-induced liver injury is a more challenge in the pediatric setting, even for short periods of exposure because of the age-dependent maturation of the cytochrome P450 enzymes involved in the antibiotic metabolism [8]. Therefore, minimizing antibiotics-associated adverse effects is particularly important.

It has been verified that antibiotic exposures can also damage the liver by disrupting the intestinal barrier, which allows live commensal gut microbiota to translocate to the liver and leads to impaired T-cell functions in the liver [9]. Moreover, communication of inflammatory mediators between the gut and the liver means that gut homeostasis dysregulation affects liver health [10]. However, studies on the harm and rescue measures of antibiotics from the perspective of the gut–liver system are lacking.

Baicalin, a kind of flavonoid extracted from the root of *Scutellaria*, has received much more attention due to its extensive pharmacological activities, including anti-inflammation, anti-oxidation, anti-apoptosis, and the protective effects on intestinal and liver disease [11]. Baicalin regulates intestinal immunity and protects against intestinal damage due to ulcerative colitis and sepsis [12,13]. Baicalin protects against several types of liver diseases including viral hepatitis, fatty liver disease, xenobiotic-induced liver injury, cholestatic liver injury, and hepatocellular carcinoma, with a variety of pharmacological mechanisms [14]. It reverses the epithelial–mesenchymal transition through the inhibition of the TGF-β1/Smad3 pathway in vitro to prevent the development of liver fibrosis [15]. However, the significance of baicalin in the repair of antibiotic-induced gut and liver injury has not been systematically reported.

In this study, through RNA-seq and 16s microbiome, we aimed at elucidating on the relevant signaling pathways and potential molecular mechanisms through which baicalin ameliorates short-term lincomycin-induced intestinal and liver injury in infant mice models. Our findings expand our knowledge on antibiotic-induced injury and provide new avenues for identifying potential therapeutic targets for treatment of antibiotic-induced injury and its complications.

## 2. Results

### 2.1. Growth Performance and Intestinal and Liver Morphologies

**Figure 1:**
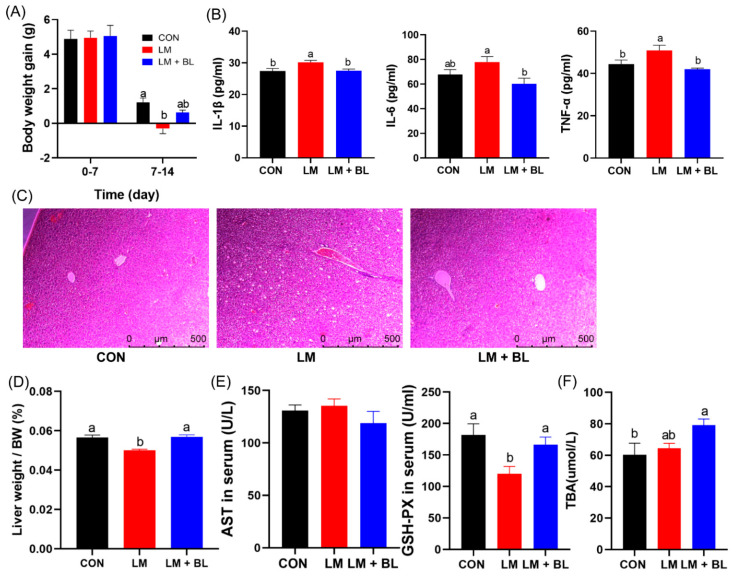
Growth performance and liver morphologies. (**A**) Body weight gain (*n* = 16). (**B**) IL-1β, IL-6, and TNF-α levels in serum (*n* = 12). (**C**) Staining profiles by H&E of liver (scale bars: 500 μm). (**D**) Liver weight/body weight. (**E**) AST and GSH-PX levels in serum (*n* = 8). (**F**) TBA levels in liver (*n* = 8). Values are means with their standard error means represented by vertical bars. Comparisons of means among groups was performed by one-way ANOVA followed by multiple comparisons using Tukey’s HSD test. Different letters indicate significant differences between the two groups (*p* < 0.05), while the same or no letter indicates insignificant differences (*p* > 0.05).

Body weight gain for the LM group was less than CON and LM + BL in the second week (*p* < 0.05, Figure 1A). Lincomycin exposure reduced the liver weight/BW, but baicalin restored the liver weight/BW to CON levels (Figure 1D, *p* < 0.05). Baicalin reduced water intake/feed intake in the second week (Figure S1A, *p* < 0.05, see Supplementary Materials). There was significant increase in serum IL-1β and TNF-α levels in the LM group compared to the CON group (*p* < 0.05); however, serum IL-1β, IL-6, and TNF-α levels in the LM + BL group were markedly decreased compared to the LM group (Figure 1B, *p* < 0.05).

Pathologic examination confirmed the therapeutic potency of baicalin in ameliorating the phenotypes of lincomycin-treated mice, namely, hepatocyte injury and ballooning (Figure 1C). In addition, glutathione peroxidase (GSH-PX) levels in serum were reduced by lincomycin but restored to control levels by baicalin (Figure 1E, *p* < 0.05). Baicalin increased total bile acid (TBA) levels in the livers of lincomycin-treated mice compared to CON (Figure 1F, *p* < 0.05).

**Figure 2:**
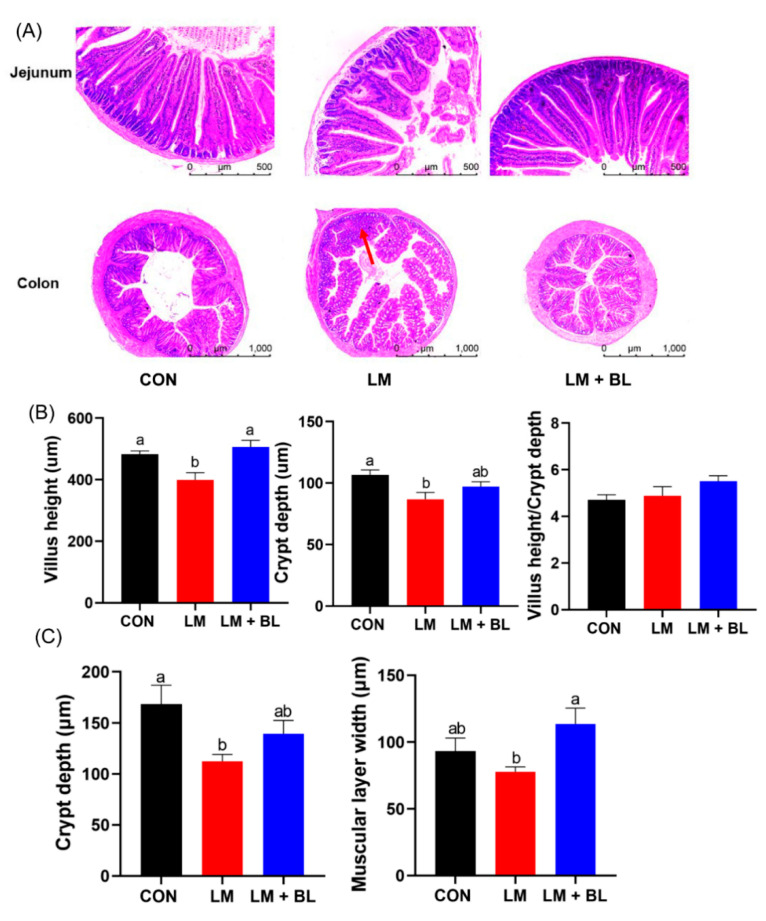
Intestinal morphologies. (**A**) Staining profiles by H&E of jejunum (scale bars: 500 μm) and colon (scale bars: 1000 μm). (**B**) Villus height, crypt depth, and villus height/crypt depth of jejunum. (**C**) Crypt depth and muscular layer width of colon. Values are means (*n* = 12/diet), with their standard error means represented by vertical bars. Comparisons of means among groups was performed by one-way ANOVA followed by multiple comparisons using the Tukey’s HSD test. Different letters indicate significant differences between the two groups (*p* < 0.05), while the same or no letter indicates insignificant differences (*p* > 0.05).

Jejunum histology was disrupted in LM group, with irregular epithelial cells, short and exfoliated villi, and shallow crypts (Figure 1D). However, these damages were improved by baicalin, as shown by regular cell arrangement in thicker and longer villi and deeper crypts. Lincomycin exposure significantly reduced jejunum villi height and crypt depth (*p* < 0.05), but baicalin restored jejunum villi height to the control level (Figure 2B, *p* < 0.05). Lincomycin exposure decreased colon crypt depth and muscular layer width (Figure 2C, *p* < 0.05). Lincomycin exposure also induced inflammatory cell infiltration (Figure 2A). Interestingly, baicalin improved colon muscular layer thickness (Figure 2C, *p* < 0.05). Baicalin also tended to increase *zonula occludens 1* (*ZO-1*) expressions in lincomycin-treated mice (Figure S1B, *p* = 0.0893), while baicalin decreased *Mucin2* expressions in lincomycin-treated mice (Figure S1B, *p* < 0.05).

### 2.2. Colon Transcriptome

**Figure 3:**
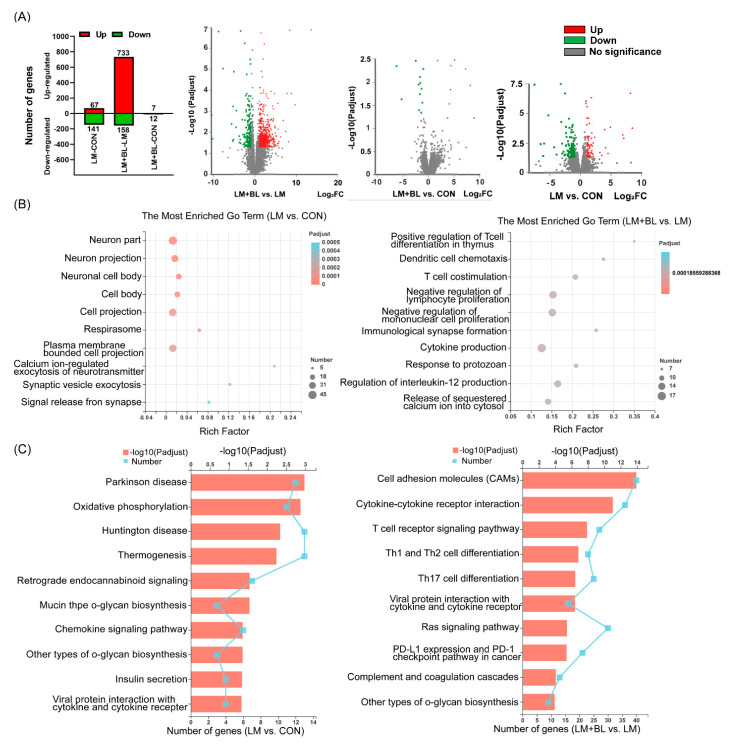
Colon transcriptome. (**A**) Upregulated (red bars) and downregulated (green bars) DEG numbers and volcano plots of identified genes by comparisons between LM + BL and LM, between LM + BL and CON, and between LM and CON (*n* = 4 in CON and *n* = 5 in LM and LM + BL). The *p*-adjust < 0.05 and absolute fold change ≥ 2 were used as the thresholds to filter the DEGs. Green spots indicate downregulated genes, while red spots indicate upregulated genes and gray ones indicate the genes that are not significant. (**B**) GO function analysis for DEGs in LM vs. CON and in LM + BL vs. LM. (**C**) KEGG pathway enrichment analysis for DEGs in LM vs. CON and in LM + BL vs. LM. The top 10 functions are shown.

There were 67 upregulated and 141 downregulated DEGs (*p*-adjust < 0.05 and absolute fold change ≥ 2) in LM vs. CON, 733 upregulated and 158 downregulated DEGs (*p*-adjust < 0.05 and absolute fold change ≥ 2) in LM + BL vs. LM, and only 7 upregulated and 12 downregulated DEGs (*p*-adjust < 0.05 and absolute fold change ≥ 2) in LM + BL vs. CON (Figure 2A). The DEGs in LM + BL vs. CON were enriched in the functions of neurons (neuron part, neuron projection, neuronal cell body), cells (cell body, cell projection, respirasome, plasma-membrane-bounded cell projection), and synapses (calcium-ion-regulated exocytosis of neurotransmitters, synaptic vesicle exocytosis, signal release), and were mainly involved in pathways of some disease (Parkinson’s disease, oxidative phosphorylation, Huntington’s disease, thermogenesis, retrograde endocannabinoid signaling), mucin and other types of O-glycan biosynthesis, and insulin secretion (Figure 3B,C). The DEGs in LM + BL vs. LM were enriched in immune functions, such as the regulation of immune cells (T cells, dendritic cells, lymphocytes, and monocytes), cytokine production, and responses to protozoans. KEGG pathway analysis showed that these DEGs were highly enriched in pathways of T-cell receptor signaling; Th1, Th2, and Th17 cell differentiation; cytokine with cytokine receptor; and viral protein interactions (Figure 3B,C). Then, the expression patterns of DEGs that both selected in LM + BL vs. LM and LM vs. CON (*p*-adjust < 0.05 and absolute fold change ≥ 2) were similar in LM + BL and CON groups, but contrary to the LM group (Figure S2A,B). These DEGs were enriched in mucin type and other types O-glycan biosynthesis pathways (Figure S2C).

### 2.3. Liver Transcriptome

**Figure 4:**
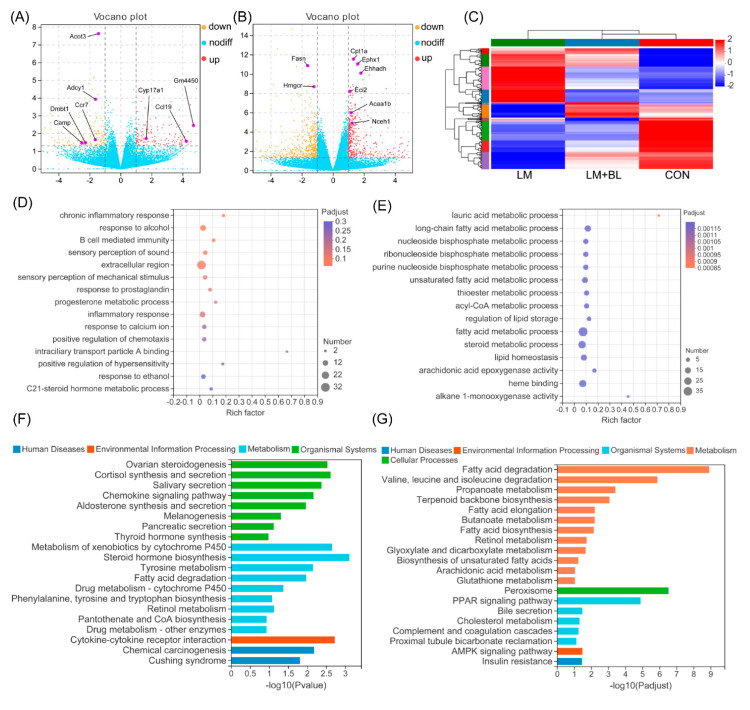
Liver transcriptome. (**A**) Volcano plots of identified genes by comparisons between LM and CON (*n* = 5), and (**B**) between LM + BL and LM. The *p*-value < 0.05 and absolute fold change ≥ 2 was used as the thresholds to filter the DEGs. Yellow spots indicate downregulated genes, while the red spots indicate upregulated genes, and blue ones indicate the genes that were not significant. (**C**) Heat map clustering for DEGs in LM vs. CON. (**D**) GO function analysis for DEGs in LM vs. CON and (**E**) in LM + BL vs. LM. The top 15 functions are shown. (**F**) KEGG pathway enrichment analysis for DEGs in LM vs. CON and (**G**) in LM + BL vs. LM.

After quality control and mapping of sequences from RNA-Seq, DEGs were filtered using the DESeq2 method. A total of 285 DEGs (131 upregulated and 154 downregulated; *p*-value < 0.05, fold-change ≥ 2) were filtered in the liver of lincomycin-treated mice (LM vs. CON) (Figure 4A). Expression patterns of the DEGs in LM + BL and CON were comparable, but contrary to LM (Figure 4C). A total of 933 DEGs were observed in LM + BL vs. CON (384 upregulated genes and 549 downregulated; *p*-value < 0.05, fold-change ≥ 2) (Figure 4B).

GO enrichment analysis for DEGs in LM vs. CON revealed that they were enriched in pathways related to B-cell-mediated immunity, inflammatory responses, and responses to alcohol (*p* < 0.05, Figure 4D). KEGG pathway analysis (Figure 4F) revealed that these DEGs were markedly enriched in pathways related to lipid synthesis (ovarian steroidogenesis; cortisol synthesis and secretion; aldosterone synthesis and secretion; fatty acid degradation; phenylalanine, tyrosine, and tryptophan biosynthesis; pantothenate and COA biosynthesis), hormone biosynthesis (thyroid hormone synthesis, steroid hormone biosynthesis, salivary secretion, and pancreatic secretion), and chemotaxis (chemokine signaling pathway, cytokine-cytokine receptor interaction). GO enrichment analysis for DEGs in LM + BL vs. LM (Figure 4E) revealed that they were markedly enriched in lipid metabolism processes (lauric acid, long-chain fatty acid, nucleoside bisphosphate, ribonucleoside bisphosphate, purine nucleoside bisphosphate, unsaturated fatty acid, thioester, acyl-CoA, and fatty acid, as well as steroid metabolic process) and lipid homeostasis (regulation of lipid storage, lipid homeostasis, arachidonic acid epoxygenase activity, heme binding, alkane 1-monooxygenase activity). KEGG pathway analysis revealed that these DEGs (Figure 4G) were enriched in pathways related to lipid degradation (fatty acid degradation; valine, leucine, and isoleucine degradation; propanoate metabolism; cholesterol metabolism), fatty acid metabolism (terpenoid backbone biosynthesis, fatty acid elongation, butanoate metabolism, retinol metabolism, peroxisome), bile secretion, and signaling pathways (PPAR and AMPK signaling pathways).

### 2.4. Validation of the RNA-Seq Results by qPCR

**Figure 5:**
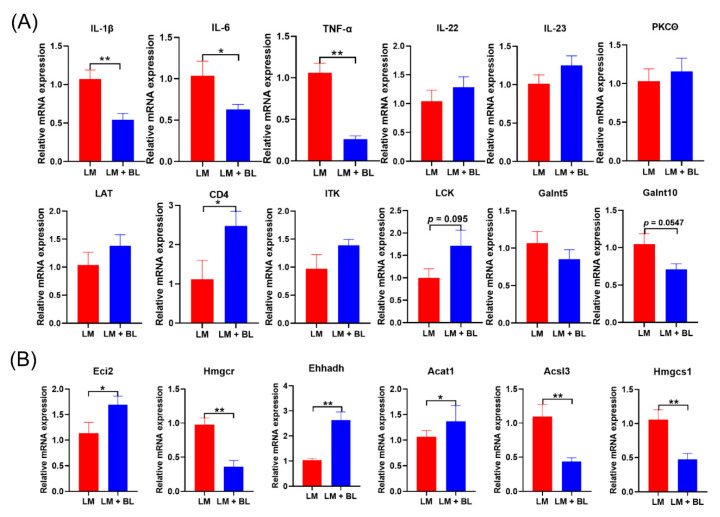
The mRNA expression of key DEGs: (**A**) Related cytokines *IL-1β*, *IL-6*, *TNF-α*, *IL-22*, and *IL-23*, and *PKCθ*, *CD4*, *LCK*, *LAT*, *ITK*, *Galnt5*, *Galnt10* in colon, and (**B**) *Eci2*, *Hmgcr*, *Ehhadh*, *Acat1*, *Acsl3*, and *Hmgcs1* in liver. Values are means with their standard error means represented by vertical bars (*n* = 10). * means *p* < 0.05, ** means *p* < 0.01.

To validate the RNA-seq results, qRT-PCR analysis was conducted for key DEGs and related cytokines in the colon and liver. The mRNA expressions of *CD4* in colons of LM + BL were significantly greater than those of LM (Figure 5A, *p* < 0.05), mRNA expressions of *LCK* in LM + BL were increased, compared to LM (*p* = 0.095), and *Galnt10* levels in the colon were decreased (*p* = 0.0547). Expressions of *IL-22*, *IL-23*, *PKCθ*, *LAT*, *ITK*, and *Galnt5* in the colon exhibited similar patterns with RNA-Seq (Figure 5A). In addition, relative mRNA expressions of *TNF-α*, *IL-1β*, and *IL-6* in the colons from the LM + BL group were significantly decreased, compared to LM (Figure 5A, *p* < 0.05). The mRNA expressions of *Eci2*, *Ehhadh*, and *Acat1* in livers were significantly increased, while *Hmgcr*, *Acsl3*, and *Hmgcs1* levels in livers of the LM + BL group were decreased, compared to LM (Figure 5B), consistent with findings from RNA-seq.

### 2.5. Colon Microbiome

**Figure 6:**
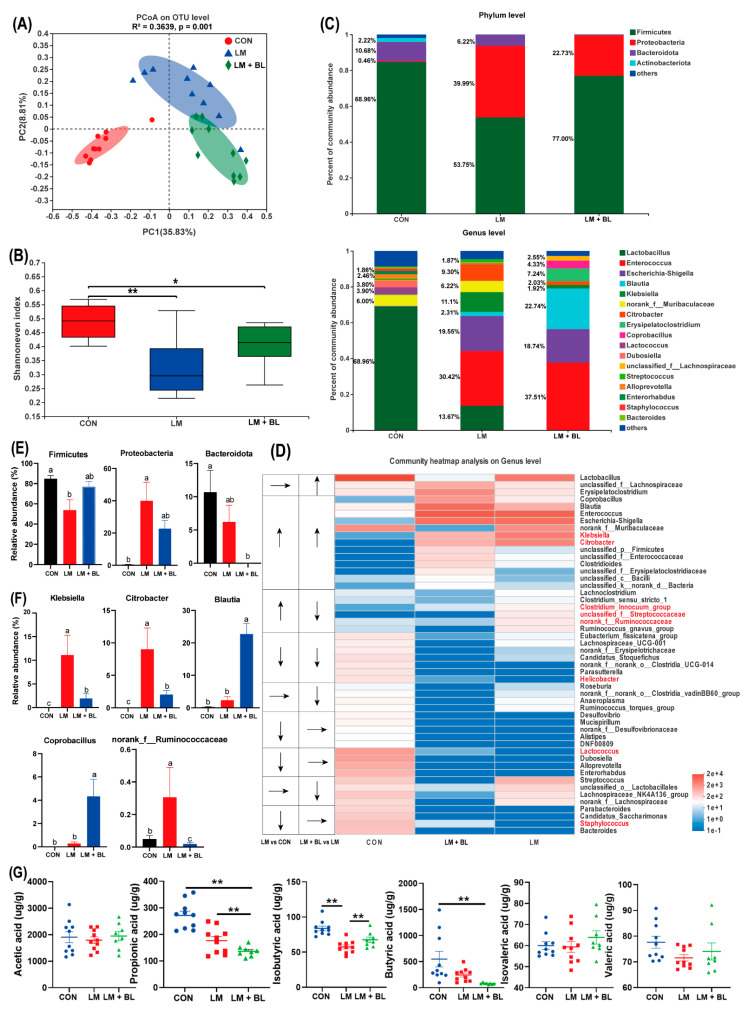
Colon microbiome. (**A**) PCoA analyze. (**B**) Alpha-diversity of fecal microbiota in colon chyme of Shannoneven index. (**C**) Relative abundance of microbiota at the phylum level and genus levels. (**D**) Heat map and trend of bacterial at the genus level. (**E**) Phylum level bacterial statistical comparison in three groups. (**F**) Genus level bacterial statistical comparison in three groups. (**G**) Scatter plot for short-chain fatty acid concentration in colon contents (*n* = 10 in CON and LM, *n* = 8 in LM + BL). Intergroup difference test was performed by Wilcoxon rank-sum test. * means statistically significant (*p* < 0.05), ** means *p* < 0.01. Less than 1% abundance of the phyla or genus was merged into others. Different letters indicate significant differences between the two groups (*p* < 0.05), while the same letter indicates insignificant differences (*p* > 0.05).

Principal coordinate analysis based on unweighted unifrac distance metric is shown in Figure 6A. Differential clustering of gut microbiota structures among CON, LM, and LM + BL groups revealed different microbial communities. Alpha diversity (Chao, Shannon, and Shannoneven indices) revealed that gut microbiota diversity in LM and LM + BL groups was significantly lower compared to CON (Figure 6B and Figure S1C, *p* < 0.01).

Microbial community compositions of the three groups are shown in Figure 6C. At the phylum level, most abundant phyla in CON were *Firmicutes* (68.96%), *Bacteroidota* (10.68%), *Actinobacteria* (2.22%), and *Proteobacteria* (0.46%). In LM, relative abundance of *Firmicutes* (53.75%) and *Actinobacteria* (6.22%) were decreased, whereas relative abundance of *Proteobacteria* (39.99%) was increased (*p* < 0.05, Figure 6E). However, relative abundance of *Firmicutes* (77.00%) in LM + BL was greater than that of LM, but the relative abundance of *Bacteroidota* and *Proteobacteria* (22.73%) was less than that of LM. At the genus level, gut microbiota composition in CON included *Lactobacillus* (68.96%), *norank_f__Muribaculaceae* (6.00%), *Lactococcus* (3.90%), *Dubosiella* (3.80%), *Alloprevotella* (2.46%), and other genera (Figure 6C). Relative abundances of *Enterococcus* (30.42%), *Escherichia-Shigella* (19.55%), and *Klebsiella* (11.1%) were increased in LM, while relative abundances of *Lactobacillus* (13.67%) were reduced. The top five genera in LM + BL were *Enterococcus* (37.51%), *Blautia* (22.74%)*, Escherichia-Shigella* (18.74%), *Eryslpelatoclostridlum* (7.24%), and *Coprobacillus* (4.33%). Alterations of the top 50 genera in relative abundance attracted more attention. Relative abundance for most genera (e.g., *Bacteroides*, *Staphylococcus*, *Candidatus_Saccharimonas*, *Parabacteroides, Lactococcus, Dubosiella*) were diminished due to lincomycin exposure (Figure 6D). For some of the genera, their relative abundances increased due to lincomycin exposure (e.g., *Clostridium_sensu_stricto_1*, *Clostridium_innocuum_group*, *UNCLASSIFIED_F__STREPTOCOCCACEAE*, *norank_f__Ruminococcaceae*, *Klebsiella*, *Citrobacter*; Figure 6D). Baicalin treatment restored normal mass for some of the genera (e.g., *Klebsiella*, *Citrobacter*, *Clostridium_innocuum_group*, *unclassified_f__Streptococcaceae*, *norank_f__Ruminococcaceae*, *Helicobacter*, *Lactococcus*, *Staphylococcus*; Figure 6D). The abundance of *Klebsiella*, *Citrobacter*, and *norank_f_Ruminococcaceae* were significantly increased in LM compared to CON, but significantly decreased after baicalin treatment compared to LM (*p* < 0.05, Figure 6F). Abundances of *Blautia* and *Coprobacillus* were significantly increased in the LM + BL group compared to LM (*p* < 0.05, Figure 6F). The expressions of DEGs that were common in LM vs. CON and LM + BL vs. LM were correlated with abundances of *Bacteroidota* and *Firmicutes* (positively) and *Lachnoclostridium*, *Streptococcus*, *Lactobacillus*, and *Lactococcus* (negatively; Figure S3).

On the basis of gut microbiota changes, quantification of SCFAs in colon chyme were performed (Figure 6G). Relative concentrations of isobutyric acid were significantly suppressed by lincomycin exposure but restored to control levels by baicalin treatment (*p* < 0.05). However, relative propionate and butyrate acid levels in LM + BL were less when compared to CON (*p* < 0.05).

## 3. Discussion

In this study, lincomycin exposure decreased bodyweight gain of mice, in tandem with a previous study [16]. This could have been due to lincomycin-induced reduction in jejunum villus height, crypt depth, and colon muscular layer width, which might have caused a reduction in contact area between nutrients and intestines as well as affected intestinal peristalsis to reduce nutrient absorption. Disruption of the intestinal barrier also led to leakage, causing liver damage and inflammation. This was confirmed by morphological changes and functional impairments of the liver after lincomycin exposure. These results may be attributed to the anti-inflammatory, antibacterial, and intestinal protection activities of baicalin [17]. However, the mechanisms through which baicalin aids in intestinal and liver injury recovery should be evaluated further.

**Figure 7:**
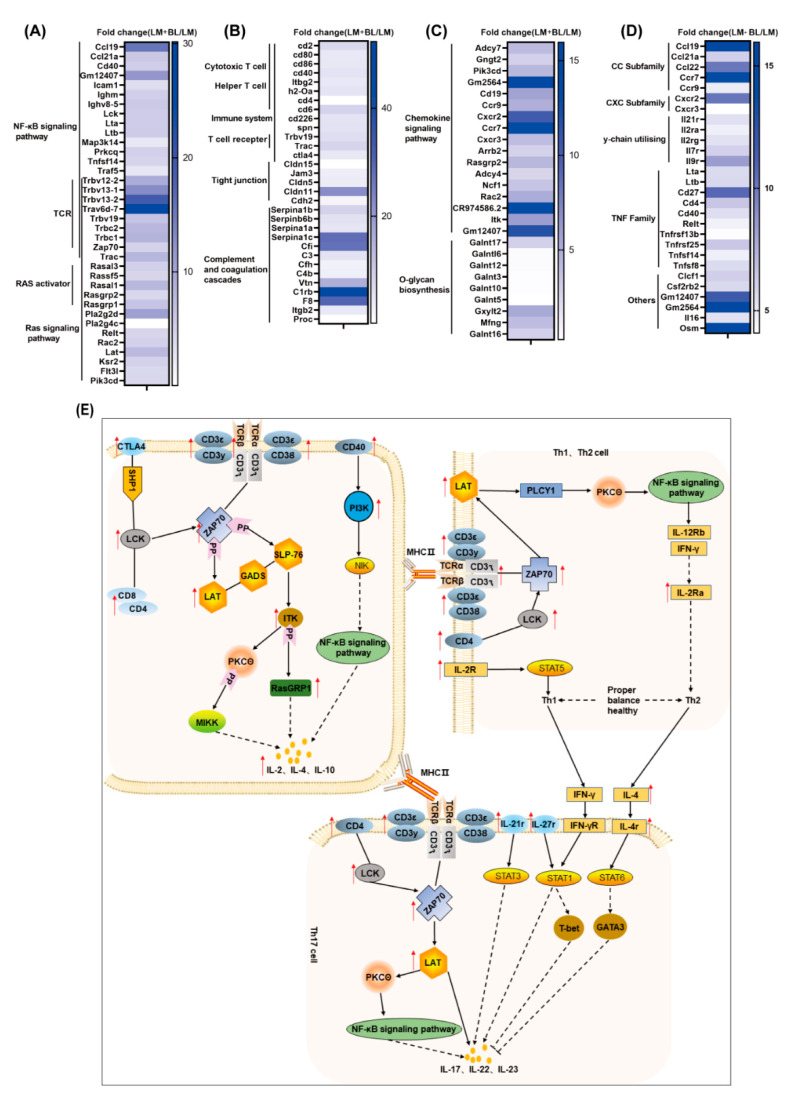
Transcriptomics revealed that baicalin regulates intestinal immunity and repairs intestinal damage. Fold change of differentially expressed genes related to (**A**) NF-κB signaling pathway and ras signaling pathway; (**B**) intestinal immunity cell adhesion molecules and complement and coagulation cascades; (**C**) chemokine signaling pathway and O-glycan biosynthesis; (**D**) cytokine–cytokine receptor interaction as well as T-cell receptor signaling pathway; and (**E**) the process of Th1/Th2 and Th17 cell differentiation. The red arrows indicate upregulation.

Efficient tissue repair after injury is indispensable for all organisms. Tissue repair processes, including inflammation, cell proliferation, differentiation, and migration, as well as cell junction, are involved in the formation of a complete mucosal barrier [18]. Inflammation, the physiological response during tissue injury, transports effector cells to damaged locations and provides a barrier to prevent infections from spreading. We found upregulated expressions of inflammatory cytokines and their receptors (*IL-16*, *IL-21r*, *IL-2ra*, *IL-2rg*, *IL-7r*, *IL-9r*, *LTA*, *LTB*, *Tnfrsf13b*, *Tnfrsf25*, *Tnfsf14*, and *Tnfsf8*; Figure 7D) in LM + BL vs. LM, suggesting that baicalin may regulate the inflammatory processes to promote intestinal repair. *IL-21*/*IL-21r* regulates intestinal inflammation by deviating from Th1/Th2 to Th2 equilibrium [19]. *IL-2* and *IL-7r* stimulate T-cell proliferation and differentiation and have positive effects on proliferations and differentiations of other cells [20,21]. The tumor necrosis factor (TNF) family, including *Tnfrsf13b, Tnfsf14, Tnfsf25,* and *Tnfsf8*, play an important role in regulation of inflammatory response and tissue homeostasis. Moreover, the TNF family is involved in cell growth, differentiation, and death [22,23,24,25]. During inflammatory processes, leukocytes ooze to inflammation areas by adhering and traversing the vascular endothelial cells [26]. Cell adhesion molecules, such as *Cd2*, *Cd80*, *Cd86*, *Cd40*, *Itbg2*, *Ctla4*, *Cd4*, *Cd6*, *Cd226*, *Spn*, *H2-Oa*, *Trbv19*, *Trac*, *Jam3*, *Cldn5*, *Cldn11*, and *Cdh2*, play important roles in this process, and all of them were upregulated in this study (Figure 7A). *Ras*, which is located in the inner side of the cell membrane, is responsible for transmitting signals from the extracellular matrix and is also involved in intercellular adhesion. After activation by cytokines, the activated ras pathway regulates cell proliferation, differentiation, apoptosis, and metabolism [27]. In this study, the genes associated with this pathway were upregulated in the LM + BL group (Figure 7A). *Cd80* and *Cd86*, which belong to the immunoglobulin superfamily, combine with the *Cd28* ligand to promote T-cell proliferation and activation [28]. *Cd40*/*Cd40L* induce endothelial cells to express intercellular adhesion molecule-1, which acts as a ligand for lymphocytes and *Itbg2*, and forms adhesion between immune cells and vascular endothelial cells [29]. The *Jam3*, *Cldn5*, *Cldn11*, and *Cdh2* are involved in tight junction formation. *Cdh2* increases the number of tight junction structural pores and enhances the transport of paracellular substances [30]. *Cdh5* reinforces connections between cells and reduces the permeability of tight junction [31]. Chemokines are beneficial in intestinal repair by taking part in immune cell migration and aggregation, especially phagocytes and lymphocytes [32]. For instance, *Cxcr2*, the *Cxcl1-8* receptor, attracts neutrophils to leave the bloodstream and migrate to surrounding tissues [33]. Chemokines, including *Ccl19*, *Ccl21a*, *Ccl22*, *Ccr7*, *Ccr9*, *Cd27*, *Clcf1*, *Csf2rb2*, *Cxcr2*, and *Cxcr3*, were upregulated in LM + BL vs. LM (Figure 7C).

Immune cells identify pathogen-associated molecular patterns released by injured cells and initiate tissue repair after intestinal tissue injury. During this process, lymphocytes rely on cell surface receptors to receive signals from damaged tissues. The T-cell receptor complex is composed of the TCR dimer, *CD3δ/ε* dimer, *CD3 γ/ε* dimer, and *CD3ζ/ζ* dimer, which efficiently transmit the received signal into the cell [34]. In this study, KEGG pathway analysis of DEGs revealed that they were enriched in the T-cell receptor signaling pathway (*TCR*, *CD3δ/ε*, *CD3 γ/ε*, *CD3ζ/ζ*, *Ctla4*, *CD40*, *PI3K*, *Rasgrp1*, *ITK*, *LAT*, *CD8*, *CD4*, *LCK*, *ZAP70*; Figure 7E). *Lck* activates *TCR* by phosphorylating *ITAMs*, the immune receptor of the *TCR/CD3* complex, and then activates *PTK* by phosphorylation of *LCK*, *FYN*, *ZAP70*, and *ITK* among others [35]. T-cell receptors recognized specific antigens and activated T cells to produce cytokines to perform immunomodulatory functions [36], such as the differentiation of Th1, Th2, and Th17 cells. The Th1/Th2 ratio maintains the balance between cellular and humoral immune responses. Imbalances in this ratio induce inflammation and diseases [37]. Th1 and Th2 cell differentiations are regulated by cytokines, antigens, and antigen-presenting cells. *IFN-γ*, *IL-2r*, and *IL-12* are the main cytokines promoting Th1 cell differentiation [38]. *IL-12* also promotes *IFN-γ* production [39]. Th2 differentiates and secretes *IL-4* under the actions of the *TCR* pathway [40], which promotes Th2 cell differentiation and inhibits Th1 cell differentiation. The DEGs involved in the above processes including *CD4*, *LCK*, *ZAP70*, *LAT*, and *IL-2ra* were upregulated in the LM + BL vs. LM groups (Figure 7B). Th17-cell-mediated immunity is important for removal of extracellular bacteria by inducing epithelial cells to release antimicrobial peptides to maintain intestinal health. Upon receiving signals presented by MHCII, the *TCR* complex binds to *ZAP70* to activate *LAT*, thereby promoting Th17-cell differentiation through the NF-κB pathway and producing cytokines such as *IL-17*, *IL-22*, and *IL-23*. *CD4* binds *LCK* to activate *ZAP70* and promote Th17-cell differentiation. *IL-21r* and *IL-27r* promote Th17 differentiation to produce *IL-22* through STAT3 and STAT1, respectively. However, *IL-4* and *IFN-γ,* secreted by Th1 and Th2 cells, respectively, inhibit Th17 cell differentiation [41]. In this study, Th17-cell-differentiation-related genes, including the *TCR* complex, *LCK*, *CD4*, *ZAP70*, *LAT*, *IL-4r*, *IL-21r*, and *IL-27r* were upregulated after baicalin treatment in lincomycin exposure mice, indicating that baicalin promotes the production of *IL-17*, *IL-22*, and *IL-23.* Elevated *IL-22* and *IL-23* mRNA expressions in LM + BL compared to LM supports this conclusion. Baicalin has been reported to regulate the balance of mouse antibody levels and expressions of Th17/Treg-associated cytokines [42]. *IL-23* is essential for maintaining Th17 cell proliferations, phenotypes, and functions [43]. *IL-17a* and *IL-17f* repair HIV-1GP40-induced intestinal epithelial dysfunction by regulating the expression of tight junction genes, and the NF-κB pathway is involved in this process [44]. In the intestine, *IL-22* levels were increased in chemically induced tissue injury and have an anti-apoptotic effect that promotes wound healing [45]. The addition of exogenous *IL-22* increased gene transcription levels of survival proteins (*bcl-2*, *bcl-xL*, *mcl-1*), anti-apoptotic molecules (*c-myc*, *cyclind1*, *rb2*, *cdk4*), and mucosal barrier protective molecules (*mucin1*, *mucin2*, *mucin10*), while the deletion of *IL-22* significantly impaired tissue repair in DSS-induced colitis mice models, and no repair occurred after acute injury [46,47]. *IL-22* inhibits *Clostridium* difficile-induced intestinal damage by activating the complement system [48]. In this study, complement activation was also observed (*Serpina1b*, *Serpinb6b*, *Serpina1a*, *Serpina1c*, *Cfi*, *C3*, *Vtn*, *C1rb*, *F8*, *Itgb2*, *Proc*, *Cfh*, *C4b*). These results suggest that baicalin enhances intestinal repair after injury by promoting Th17 cell differentiation and *IL*-*22* production, consistent with findings from a previous study that baicalin regulates Th1, Th17, and Treg responses to ameliorate sepsis-associated pancreatic injury through the ras homolog family member A -Rho kinase pathway [13].

**Figure 8:**
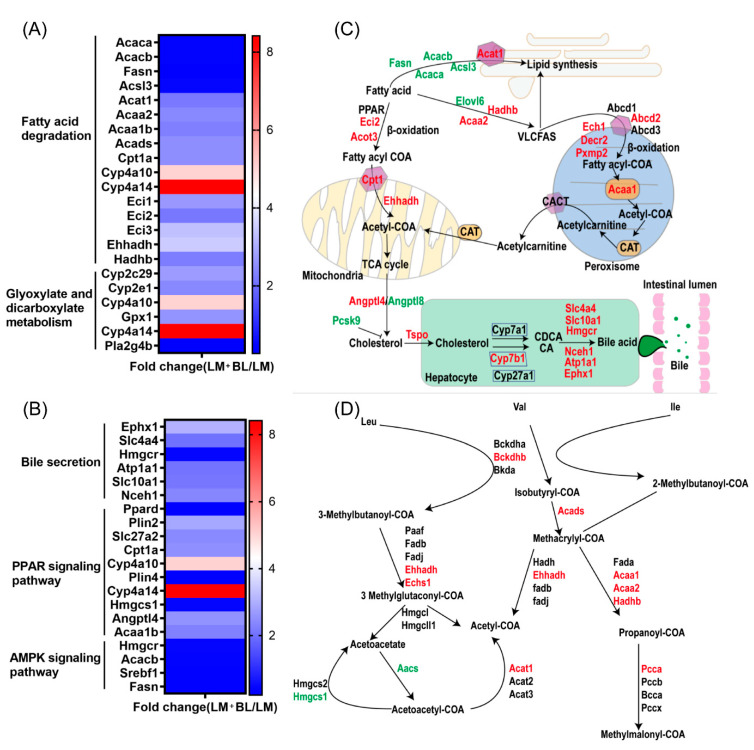
Transcriptomics revealed that baicalin regulates lipid metabolism and bile secretion. Fold change of differential expressed genes related to (**A**) fatty acid degradation and glyoxylate and dicarboxylate metabolism, and (**B**) bile secretion, PPAR signaling pathway, and AMPK signaling pathway. (**C**) The process of fatty acid degradation, peroxisome, fatty acid biosynthesis, and bile secretion. (**D**) The process of glyoxylate and dicarboxylate metabolism. The red words indicate upregulation and green words indicate downregulation.

Intestinal mucosal damage breaks the barrier between intestines and blood, thereby increasing permeability. This allows entry of bacteria and their metabolites into the blood circulatory system, which increases the risk of inflammation and liver damage. Liver injury is a common extra intestinal manifestation of IBD [49]. The liver is the major organ for lipid metabolism, and disturbances in lipid metabolism is one of the causes of liver injury [50]. The downregulated DEGs in the liver of the LM group were involved in lipid metabolism pathways, including “fatty acid degradation” and “pantothenate and CoA biosynthesis” The DEGs in the liver LM group were enriched in immune- and inflammatory-response-related pathways suggested reduced ability of the liver to utilize lipids and inflammation, which may lead to lipid accumulation. The upregulated DEGs in the liver of the LM + BL group were involved in lipid metabolism pathways including “fatty acid degradation”; “valine, leucine, and isoleucine degradation”; “arachidonic acid metabolism”; “peroxisome”; “PPAR signaling pathway”; and “AMPK signaling pathway”. The downregulated DEGs in the liver of the LM + BL group were involved in “fatty acid elongation”, “fatty acid biosynthesis”, and “biosynthesis of unsaturated fatty acids” (Figure 8A,B). The aforementioned changes in these pathways suggested increased ability of the liver to utilize lipids that will decrease lipid synthesis and lipid accumulation. PPAR is an important signal pathway that regulates the oxidative decomposition of fatty acids [51]. As an energy sensor, AMPK maintains the balance between fat content and lipid metabolism, and its activation increased energy decomposition [52]. *Fasn*, *Acacb*, and *Acaca* are involved in fat synthesis, and *Acsl3* promotes the formation of intracellular lipid droplets, hence influencing fat deposition [53,54]. The downregulated expression of these genes in the LM + BL group suggested that baicalin ameliorated antibiotic-induced fat deposition. *Acot3*, *Eci2*, *Ehhadh*, *Acaa2*, *Hadhb*, and *Cpt1* are genes involved in fatty acid oxidation. Upregulation of these genes in the LM + BL group indicated that baicalin treatment promoted fatty acid decomposition (Figure 8C). In addition, genes involved in oxidation of very long chain fatty acids (VLCFAs) in peroxisomes such as *Abcd2*, *Ech1*, *Decr2*, *Pxmp2*, and *Acaa1* were upregulated, which further demonstrated that baicalin promoted lipolysis. Consistent with these results, a previous study showed that baicalin activated hepatic *Cpt1* to ameliorate diet-induced obesity and hepatic steatosis [55]. Genes involved in leucine, valine, and isoleucine metabolism such as *Bckdhb*, *Ehhadh*, *Echs1*, *Acads*, *Acaa1*, and *pcca* were upregulated in that LM + BL group, which enhanced acetyl-COA and methylmalonyl-COA production (Figure 8D). In liver cells, cholesterol generated from the oxidation of fatty acids is converted to bile acids through the classical and alternative pathways and secreted into the intestine to regulate intestinal peristalsis, mucus secretion, and immunity (Figure 8C). This process relies on liver-induced cytochrome P450, Cyp7b1, Cyp7a1, and Cyp27a1 to produce CA and CDCA. The expression of *Cyp7b1* determines the ratio of CA to CDCA by promoting CA biosynthesis. In this study, the expression of *Cyp7b1* and related DEGs involved in this process such as *Slc4a4*, *Slc10a1*, *Hmgcr*, *Nceh1*, *Atp1a1*, *Ephx1*, and *Tspo* was higher in the LM + BL group than the LM group, indicating increased bile acid synthesis in the LM + BL group. Bile acids are important molecules that modulate immune responses. Bile acid metabolites have been reported to act on dendritic cells to reduce their immune stimulating-effects, thereby increasing Foxp3 expression and inducing Treg cell production, which maintains intestinal mucosal immune response [56]. By screening bile acid metabolites, Jun et al. [57] found that bile acid metabolites directly regulate the balance between Th17 and Treg cells, and hence the host immunity. Impaired bile acid metabolism may induce liver inflammation and liver fibrosis. These results suggest that baicalin regulates intestinal immunity by promoting bile acid synthesis and secretion in liver.

Intestinal microbes may be intermediate regulators of liver-intestinal communication. Bile acids synthesized by liver alters the structure of bacterial communities and can inhibit the growth of specific bacteria, which affects the metabolism and immunity of the host [58]. Atarashi et al. [59] found that *Blautia* accelerated the recovery of intestinal inflammation in mice by increasing infiltration of the large intestine with regulatory T cells, which improve outcomes of colorectal cancer and liver cirrhosis. In this study, the *Blautia* abundance was increased in the LM + BL group. Gut microbiota metabolize bile acids, leading to the production of secondary bile acids [60]. For example, the gut dysbiosis seen in IBD patients decreases microbial bile acid biotransformation, which increases the levels of conjugated primary bile acids and decreases the production of secondary bile acids [61]. *Bacteroides*, *Clostridium*, *Lactobacillus*, *Bifidobacterium*, *Listeria*, and other microorganisms participate in the metabolism of bile acids in the gut [62]. In this study, antibiotic exposure resulted in intestinal microbiome disorder and increased the proportion of pathogenic bacteria. Abnormal intestinal flora leads to loosening of tight junctions and alter the integrity of mucus layers, allowing pathogens and endotoxins to enter the liver. These endotoxins enter the liver, releasing arachidonic acid products and inflammatory factors such as TNF-α, IL-1β, and IL-6. Therefore, we examined the arachidonic acid metabolism pathway in the LM + BL group. Hepatogenic inflammatory cytokines downregulate the production of bile in liver cells and aggravate the disruption of intestinal microecology, forming a vicious cycle [63]. In this study, baicalin inhibited the abundance of *Proteobacteria*, which was considered to be the main pathogenic bacteria releasing endotoxins [64] in order to counteract the lincomycin-induced increase in *Klebsiella* and *Citrobacter*, which were reported to cause intestinal disease [65,66]. These results provide evidence that baicalin inhibits the growth of intestinal pathogens, promotes the synthesis and secretion of bile acids in liver cells, regulates intestinal immune balance, and maintains liver-intestinal circulation.

In summary, the results indicate that lincomycin exposure causes intestinal and liver injury and inflammation in mice, but baicalin treatment alleviates the lincomycin-induced intestinal and liver injury. This is because baicalin alters gut microbiota dynamics and regulates the balance of Th1, Th2, and Th17, especially the differentiation of Th17, which contributes to the production of IL-22. Furthermore, baicalin influences the expression of genes involved in the regulation of immune response in intestines, lipid metabolism, and bile acid secretion pathways in the liver. These results reveal the importance of baicalin in the management of gastrointestinal and liver complications associated with lincomycin and prophylactic treatment of related antibiotics. In addition, the effects of baicalin on the complex consequences of long-term antibiotic exposure are worth further study in the future.

## 4. Materials and Methods

### 4.1. Mice and Treatment

Forty-eight 21-day-old female ICR mice obtained from the School of Medicine, Peking University (Beijing, China), were housed in the animal facility of the Institute of Animal Science under controlled conditions (lighting cycle with light from 8:30 a.m. to 8:30 p.m. and temperature at 20 ± 5 °C). After 7 days of acclimatization, mice were randomized into three groups: control (CON), lincomycin (LM), and the lincomycin and baicalin (LM + BL) groups of 16 mice each. Mice in the LM and LM + BL groups were administered with 5 g/L lincomycin in drinking water for 1 week. In the second week, mice in the LM + BL group were administered with 500 mg/kg baicalin in feed, while mice in the LM group received control feed without baicalin. Mice in the control group received drinking water and control feed for 2 weeks. Lincomycin was obtained from Beijing Solarbio Technology Co., Ltd., with a purity of 850 μg/mg and was stored at 2–8 °C. The dose of lincomycin was selected according to our previous study [5]. Baicalin and its dose was provided by Beijing Center Technology Co., LTD, with a purity > 85%. All mice had free access to food and drinking water. Water containers and feeds were changed once every three days to supply fresh antibiotics and feed. During the experimental period, body weights and food and water intakes were monitored for each animal every week. At the end of the experiment (week 2), the liver, jejunum, colon, and colonic chyme were excised. All samples were either fixed in 4% paraformaldehyde or snap-frozen in nitrogen and stored at −80 °C until processing.

### 4.2. 16S rRNA Gene Sequencing Analysis

Microbial DNA was extracted using the E.Z.N.A.^®^ soil DNA Kit (Omega Bio-tek, Norcross, GA, USA), as instructed by the manufacturer. As previously described [67], hypervariable regions (V3-V4) of the bacterial 16S rRNA gene were amplified with primer pairs 338F (5′-ACTCCTACGGGAGGCAGCAG-3′) and 806R (5′-GGACTACHVGGGTWTCTAAT-3′) using an ABI Gene Amp^®^ 9700 PCR thermocycler (ABI, Foster City, CA, USA) [68]. Meta-genomic sequencing was performed on the basis of the Illumina platform using Miseq PE300. Raw reads were deposited into the NCBI Sequence Read Archive (SRA: PRJNA765910) database.

Raw sequences were processed using the Majorbio I-Sanger Cloud Platform (www.i-sanger.com, Majorbio, Shanghai, China, accessed on 20 March 2022), and chimeric sequences were removed. Operational taxonomic units (OTUs) with a 97% similarity cutoff were clustered using UPARSE (version 7.1, http://drive5.com/uparse/, accessed on 20 March 2022). Taxonomy for each OTU representative sequences were matched to the 16S rRNA database (Silva 138) with a confidence threshold of 0.7 using an RDP Classifier (http://rdp.cme.msu.edu/, accessed on 20 March 2022). Mothur b.1.30.1 [69] was used for refraction analysis, alpha-diversity analysis, and unweighted principal coordinate analysis (PCoA). Sobs, Shannon, and Chao indices were used to evaluate community diversity. Rarefaction curves indicated that the sequencing was deep enough to capture most of the operational units within our samples.

### 4.3. Transcriptome Analysis

Total RNA from mice colon and liver were extracted by the phenol chloroform method. RNA sequencing was performed by Majorbio company (Shanghai, China), and quality control was performed to obtain high-quality RNA samples (OD260/280 = 1.8–2.2, OD260/230 ≥ 1.0, RIN ≥ 6.5, 28S:18S ≥ 1.0, > 10 μg) to construct the sequencing library as described by Zhang et al. [70]. Raw data were submitted to the NCBI Sequence Read Archive (SRA: PRJNA765945 and PRJNA823501).

Differential gene expression analysis was performed by DESeq2 software with default parameters LM vs. CON and LM + BL vs. LM. Then, GO and KEGG enrichment analyses of differentially expressed genes (DEGs) were conducted on a Majorbio I-Sanger Cloud Platform (www.i-sanger.com, accessed on 20 March 2022), with the Benjamini–Hochberg method used to adjust the *p*-value. The correlation network diagram was generated by overlapping DEGs shared by LM vs. CON and LM + BL vs. LM. The correlation relationship between overlapping DEGs and the top 50 most abundant bacteria at the genus level was calculated by Spearman’s rank correlation coefficient.

### 4.4. Relative Concentrations of Short-Chain Fatty Acids

Colon chyme samples, approximately 0.1 g of each mouse, were suspended in 1 mL ddH2O in a 1.5 mL screw-capped tube for short-chain fatty acid (SCFA) composition analysis by gas chromatography (GC), as described by Wu et al. [71].

### 4.5. Intestinal and Hepatic Morphology

Liver, jejunum, and colon samples were collected according to Xu et al. [72] and perfusion-fixed in formalin for 24 h for intestinal morphology evaluation. Tissue slides were prepared as descried by Fan et al. [73]. Briefly, the liver, jejunum, and colon were sliced into 5 μm thick sections and stained with hematoxylin and eosin (H&E). Villous height and crypt depth from all well-oriented crypt-villi units per sample were measured in the jejunum, and the ratio of villous height/crypt depth was calculated. Then, crypt depths and muscular layer width in the colon were measured.

### 4.6. Gene Expression Related to Tight Junction Proteins and Inflammatory Cytokines

Total RNA for colon tissue was extracted using the TRIzol reagent (Ambion, UT, USA) and transcribed into cDNA using a gDNA Eraser provided by the PrimeScript™ RT reagent kit (Cat # RR047A, Takara, Shige, Japan). Quantitative real-time RT-PCR (qRT-PCR) was performed using TB Green Premix Ex Taq (TaKaRa, Kusatsu, Japan). Tight junction protein genes (*Occludin*, *ZO-1*, and *Claudin-1*), inflammatory cytokines genes (*TNF-α*, *IL-1β*, and *IL-6*), selected DEGs (*IL-22*, *IL-23*, *PKCθ*, *CD4*, *LCK*, *LAT*, *ITK*, *Galnt5*, *Galnt10, Eci2, Hmgcr, Ehhah, Acat1, Acsl3,* and *Hmgcs1*), and *Mucin1* and *Mucin2* were analyzed. Primers (Table 1) used for qRT-PCR were purchased from Sangon Biotech (Shanghai, China). All results of the target genes were normalized to the housekeeping gene, *GAPDH*.

### 4.7. Inflammatory Cytokines and GSH-PX, AST in Serum, and TBA in Liver

Blood samples were collected from mice in the CON, LM, and LM + BL groups by eyeball extraction. After standing at room temperature, serum was obtained, centrifuged at 4 °C 3000 r/min for 10 min, separated, and stored at −80 °C. Serum levels of IL-1β, IL-6, and TNF-α were measured using commercially available ELISA kits (Multiskan MK3, Thermo Fisher Scientific, Waltham, NC, USA), and GSH-PX and AST were determined according to a kit from the Nanjing Jiancheng Bioengineering Institute (Nanjing, China). Mouse liver was mixed with physiological saline and centrifuged at 2500 rpm and 4 °C for 10 min. Then, the upper tissue homogenate was obtained, after which TBA levels were determined according to kit instructions.

### 4.8. Statistical Analysis

Quantitative data are expressed as mean ± standard error of the mean (SEM). The SAS 9.4 software (SAS 9.4, Institute, Cary, NC, USA) was used for all analyses. Comparisons of means among groups was performed by one-way ANOVA followed by multiple comparisons using Tukey’s HSD test. Significance was established at *p* ≤ 0.05.

## Figures and Tables

**Table 1:** Primers Used for qRT-PCR analysis.

Gene	Forward Nucleotide Sequence Primers (5′-3′)	Reverse Nucleotide Sequence Primers (5′-3′)	Product Size (bp)
*GAPDH*	ACCACAGTCCATGCCATCAC	TCCACCACCCTGTTGCTGTA	172
*IL-1β*	TCGCAGCAGCACATCAACAAGAG	AGGTCCACGGGAAAGACACAGG	97
*IL-6*	AGAGGATACCACTCCCAACAGACC	AGCCACTCCTTCTGTGACTCCAG	97
*TNF-α*	GGACTAGCCAGGAGGGAGAACAG	GCCAGTGAGTGAAAGGGACAGAAC	103
*Claudin-1*	AGATACAGTGCAAAGTCTTCGA	CAGGATGCCAATTACCATCAAG	86
*ZO-1*	CTGGTGAAGTCTCGGAAAAATG	CATCTCTTGCTGCCAAACTATC	97
*Occuldin*	TGCTTCATCGCTTCCTTAGTAA	GGGTTCACTCCCATTATGTACA	155
*Mucin1*	ACGTGAAGTCACAGCTTATACA	AGGGCAAGGAAATAGACGATAG	193
*Mucin2*	TGCTGACGAGTGGTTGGTGAATG	TGATGAGGTGGCAGACAGGAGAC	137
*IL-22*	GCAGATAACAACACAGATGTCC	GTCTTCCAGGGTGAAGTTGAG	111
*IL-23*	CAGCGGGACATATGAATCTACT	TTGAAGATGTCAGAGTCAAGCA	184
*PKC* *Ɵ*	CGCCGACAGAGCACTCATCAAC	CAGTTCCCAGAGAGAAGGCAAATCC	144
*CD4*	CAGCATGGCAAAGGTGTATTAA	GACTGAAGGTCACTTTGAACAC	202
*LCK*	AGGGAGAAGTGGTGAAACATTA	AATCCGGGAAAAGTGATACGAG	85
*LAT*	CAGCAGAATTCAGATGATGAGC	TTCACGTAATCTTCACACGACT	200
*ITK*	CTACCTGGAAAAAGCTTGTGTC	CGTGGAGCTGGTATATTGATCA	136
*Galnt5*	ACGCAGGCAGAGAGTGACAGG	GCAACAGCAGCAGCAGTAGGAG	114
*Galnt10*	CCCAACACCAGCATCATCATCCC	GACCAACACAATCTCCGCCACTAG	117
*Eci2*	CTTGTCACTGAAGTTTTCCCTG	CTTTGGTTTTCTGGAGACGAAG	234
*Hmgcr*	GGTGCAAAGTTCCTTAGTGATG	GAATAGACACACCACGTTCATG	112
*Ehhadh*	GAATGGACTCCAGAAAGCTAGT	CGCTGTATTTCATCTACCAAGC	150
*Acat1*	AGGTAGCTCAGTCGGTAGTGTTGG	TGGATCGTTTCTCGGTTCGTTTCTC	138
*Acsl3*	CGGAAATCATGGATCGGATCTA	GTGGAGTACTACACCCTTTTGA	132
*Hmgcs1*	AAGCACAGCCACCGAGCATATTC	ACCATCCCACCCCACACTGAAG	150

## Data Availability

Raw reads for 16S rRNA gene sequencing were deposited into the NCBI Sequence Read Archive (SRA: PRJNA765910) database. Raw data for transcriptome were submitted to the NCBI Sequence Read Archive (SRA: PRJNA765945 for colon, and PRJNA823501 for liver).

## Supplementary Materials

The following supporting information can be downloaded at https://www.mdpi.com/article/10.3390/ijms23116072/s1.
