# Supplementary material for: Baicalin Alleviates Short-Term Lincomycin-Induced Intestinal and Liver Injury and Inflammation in Infant Mice
Source: Int J Mol Sci. 2022 May 28;23(11):6072. doi: 10.3390/ijms23116072 (PMC9181170; doi:10.3390/ijms23116072)
Supplement: Supplementary file 1 [file ijms-23-06072-s001.zip › ijms-1719379-supplementary.pdf]

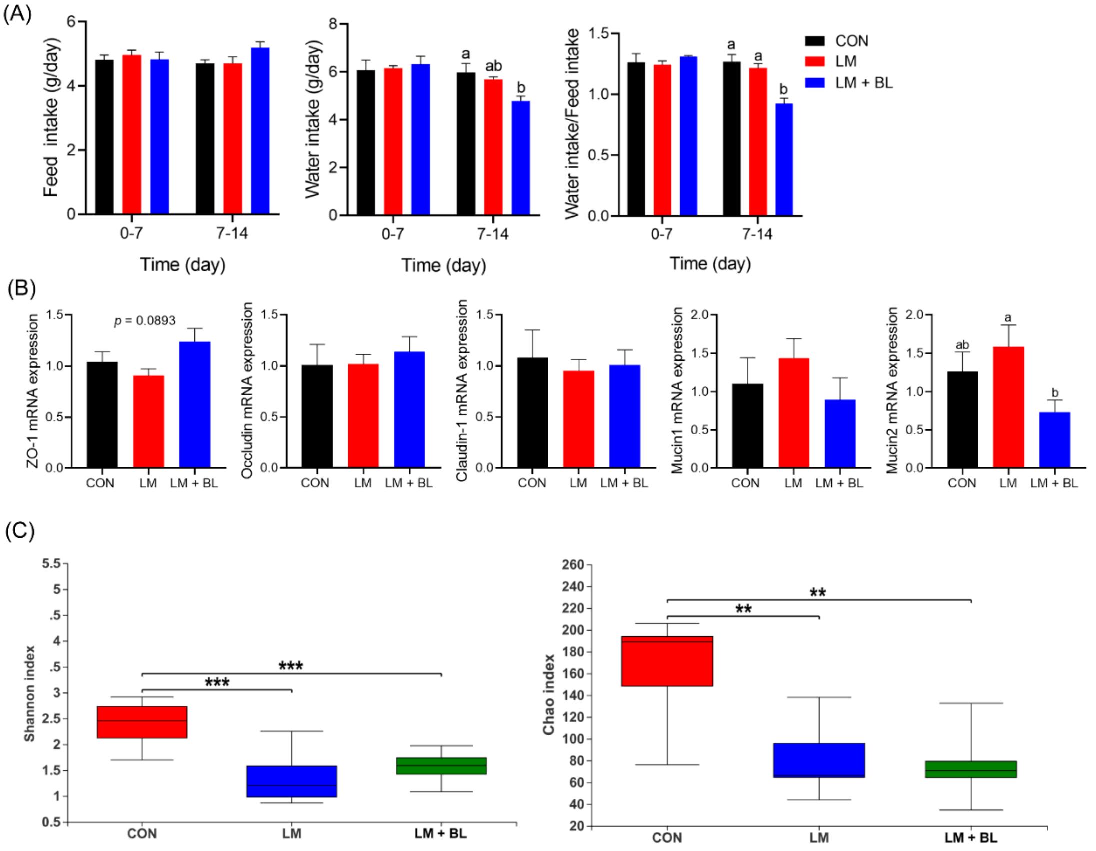

Figure S1. **(A)** Feed intake, water intake, and water intake/feed intake (n = 4). **(B)** Relative mRNA expression of *ZO-1*, *Occludin*, *Claudin-1*, *Mucin1* and *Mucin2* in colon (n = 12). **(C)** Shannon and Chao index. Values are means, with their standard error means represented by vertical bars. Comparisons of means among groups was performed by one-way ANOVA followed by multi-ple comparisons using the Tukeys' HSD test. Different letters indicate significant differences between the two groups ($p < 0{\cdot}05$), while the same or no letter indicates insignificant differences ($p > 0{\cdot}05$).

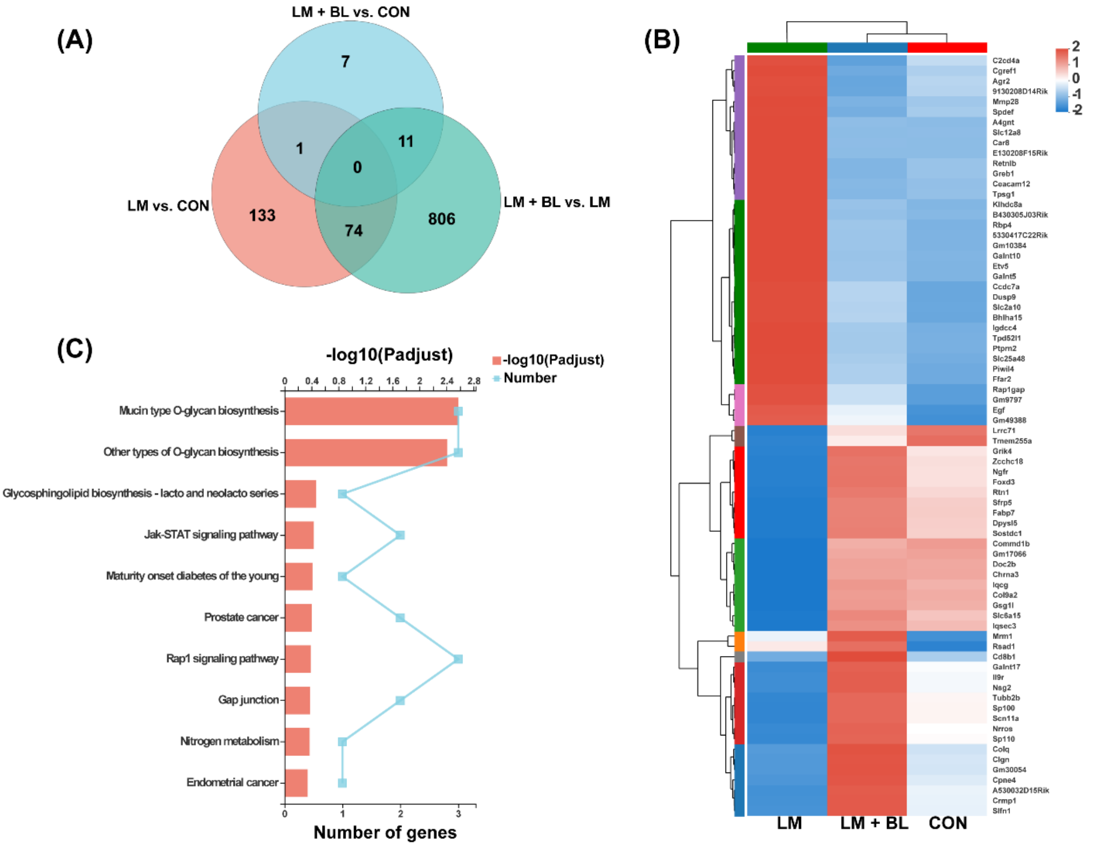

Figure S2. Changes in the colonic transcriptome after lincomycin and baicalin treatment in mice. (**A**) Venn diagram of differentially expressed genes in the LM vs. CON, LM + BL vs. LM, and LM + BL vs. CON. (**B**) Heatmap of the colonic gene expression levels in CON, LM, and LM + BL. The colors represent the relative log intensities of the gene expression levels normalized to a reference sample. (**C**) KEGG pathway enrichment analysis of the common genes of the DEGs in LM + BL compared with LM, and LM compared with CON. The top 10 pathways are shown.

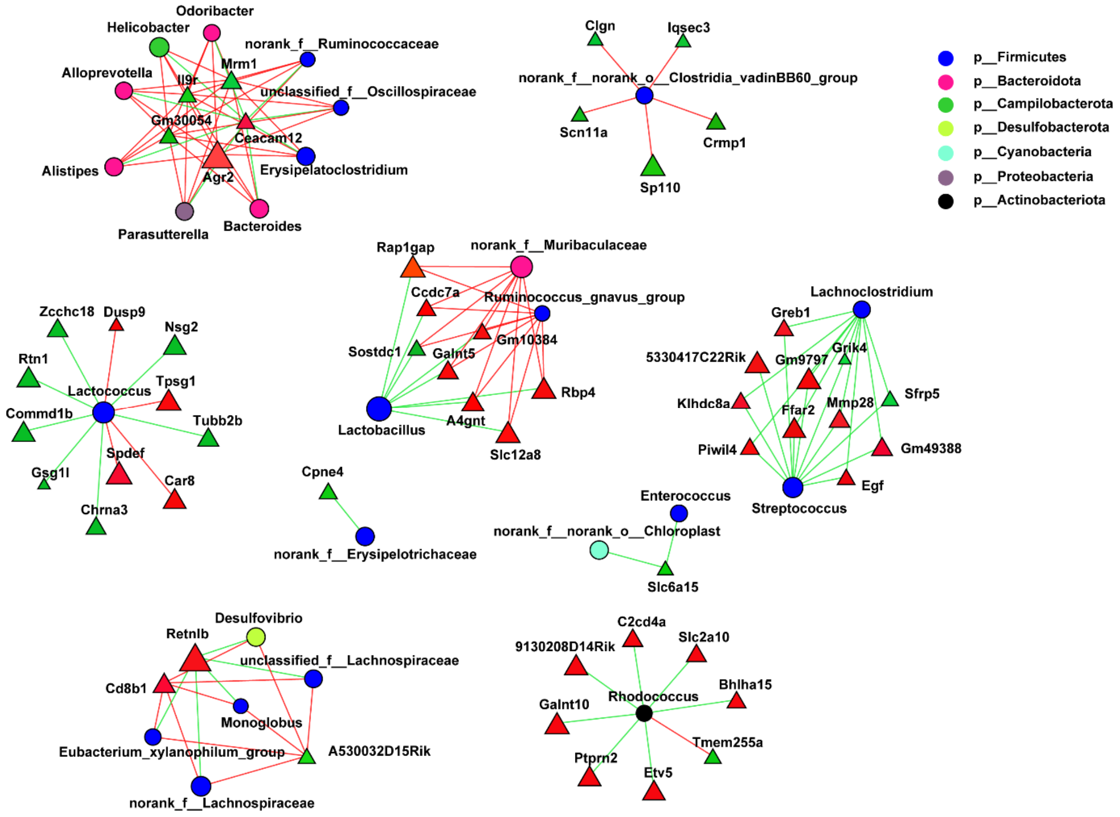

Figure S3. A network depicting correlations among microbes and DEGs in LM + BL vs. LM and LM vs. CON. Only correlations with a partial spearman's coefficient > 0.7 and a *p* < 0.05 are shown. Bacterial nodes (roundness) are OTUs labeled with their taxonomic classification. The edge color intensity indicates the level of the correlation: red, positive; green, negative. The triangular nodes represent DEGs, where the DEGs up-regulated in LM vs. CON and down-regulated in LM + BL vs. LM are in red, and the DEGs down-regulated in LM vs. CON and up-regulated in LM + BL vs. LM are in green.
